# RAGE gene polymorphism and expression: risk factor for vascular complications in type 2 diabetes mellitus

**DOI:** 10.1186/1755-8166-7-S1-P29

**Published:** 2014-01-21

**Authors:** Diwesh Chawla, Savita Bansal, Pawan K Kare, Basu Basu Dev, Sri Venkata Madhu, Ashok K Tripathi

**Affiliations:** 1 Department of Biochemistry and Department of Medicine, University College of Medical Sciences and G.T.B. Hospital, Delhi-110095, India

## Background

Advanced glycation end products (AGEs) are formed due to hyperglycemia in T2DM. Interaction of AGEs with its receptor RAGE induces signal transduction that culminates in vascular complications, the major cause of morbidity and mortality in diabetic subjects. Some functional polymorphism of this gene show differential activity of this receptor and therefore may be associated with the development of diabetic complications. In the present study we investigated the association of expression of RAGE gene and its polymorphism namely -374T/A and -429T/C in the promoter region and Gly82Ser polymorphism in the exon 3 region with vascular complications in T2DM patients.

## Materials and methods

We screened 820 subjects which includes 200 healthy controls, 200 type 2 diabetes mellitus (T2DM) subjects without any vascular complications (DM), 220 T2DM subjects with microvascular complications (DM-Micro) and 200 T2DM subjects with macrovascular complications (DM-Macro) for -374 T/A, -429 T/C and Gly82Ser polymorphisms of RAGE gene. DNA isolated from the enrolled subjects was genotyped by PCR-RFLP. RAGE expression was determined by quantitative real-time PCR.

## Results

Mutant variant of -429T/C and Gly82Ser RAGE polymorphism was about three times more prone to develop macrovascular and microvascular complications respectively in T2DM subjects while -374A allele showed reduced risk towards the development of macrovascular complications (OR = 0.57, p = 0.006). Further, haplotype analysis revealed that CTG haplotype was significantly associated with the development of macrovascular complications while haplotype TAG was observed to be significantly protective towards development of macrovascular complications in T2DM subjects. The expression of RAGE correlated significantly with the genotypic variation of the RAGE gene.

## Conclusions

Mutant genotypes of RAGE gene and its enhanced expression may be considered as risk factor for vascular complications in North Indian T2DM patients.

